# Liposomal IR-780 as a Highly Stable Nanotheranostic Agent for Improved Photothermal/Photodynamic Therapy of Brain Tumors by Convection-Enhanced Delivery

**DOI:** 10.3390/cancers13153690

**Published:** 2021-07-22

**Authors:** Yu-Jen Lu, Anilkumar T. S., Chi-Cheng Chuang, Jyh-Ping Chen

**Affiliations:** 1 Department of Neurosurgery, Chang Gung Memorial Hospital, Linkou, Kwei-San, Taoyuan 33305, Taiwan; luyj@cgmh.org.tw (Y.-J.L.); kumar@cgmh.org.tw (A.T.S.); ccc2915@cgmh.org.tw (C.-C.C.); 2 College of Medicine, Chang Gung University, Kwei-San, Taoyuan 33302, Taiwan; 3 Center for Biomedical Science and Engineering, National Tsing Hua University, Hsinchu 300044, Taiwan; 4 Department of Chemical and Materials Engineering, Chang Gung University, Kwei-San, Taoyuan 33302, Taiwan; 5 Department of Plastic and Reconstructive Surgery and Craniofacial Research Center, Chang Gung Memorial Hospital, Linkou, Kwei-San, Taoyuan 33305, Taiwan; 6 Research Center for Food and Cosmetic Safety, Research Center for Chinese Herbal Medicine, College of Human Ecology, Chang Gung University of Science and Technology, Taoyuan 33305, Taiwan; 7 Department of Materials Engineering, Ming Chi University of Technology, Tai-Shan, New Taipei City 24301, Taiwan

**Keywords:** nanomedicine, cancer therapy, brain tumor, photothermal therapy, photodynamic therapy, liposome, IR-780, convection enhanced delivery

## Abstract

**Simple Summary:**

To improve the use of hydrophobic photosensitizer IR-780 in photothermal/photodynamic therapy (PTT/PDT), we entrap IR-780 within the lipid bilayer of liposomes (ILs). Compared to free IR-780, ILs showed well-preserved photothermal response by maintaining the photostability of IR-780 from repeated near infrared (NIR) laser exposure both in vitro and in vivo. Combined with fast endocytosis by human glioblastoma cells, ILs demonstrated enhanced cytotoxicity and induced higher cell apoptosis rate toward human glioblastoma cells over free IR-780, due to PTT with overexpression of heat shock protein and PDT with generation of intracellular reactive oxygen species. To overcome the blood–brain barrier, we used convection enhanced delivery (CED) for specific delivery of ILs to brain tumors in intracranial glioma xenograft. Upon three successive NIR laser irradiations, the liposomal IR-780 could significantly improve the anti-cancer efficacy in glioma treatment, leading to diminished intracranial tumor size and prolonged animal survival time.

**Abstract:**

As a hydrophobic photosensitizer, IR-780 suffers from poor water solubility and low photostability under near infrared (NIR) light, which severely limits its use during successive NIR laser-assisted photothermal/photodynamic therapy (PTT/PDT). To solve this problem, we fabricate cationic IR-780-loaded liposomes (ILs) by entrapping IR-780 within the lipid bilayer of liposomes. We demonstrate enhanced photostability of IR-780 in ILs with well-preserved photothermal response after three repeated NIR laser exposures, in contrast to the rapid decomposition of free IR-780. The cationic nature of ILs promotes fast endocytosis of liposomal IR-780 by U87MG human glioblastoma cells within 30 min. For PTT/PDT in vitro, ILs treatment plus NIR laser irradiation leads to overexpression of heat shock protein 70 and generation of intracellular reactive oxygen species by U87MG cells, resulting in enhanced cytotoxicity and higher cell apoptosis rate. Using intracranial glioma xenograft in nude mice and administration of ILs by convection enhanced delivery (CED) to overcome blood-brain barrier, liposomal IR-780 could be specifically delivered to the brain tumor, as demonstrated from fluorescence imaging. By providing a highly stable liposomal IR-780, ILs significantly improved anti-cancer efficacy in glioma treatment, as revealed from various diagnostic imaging tools and histological examination. Overall, CED of ILs plus successive laser-assisted PTT/PDT may be an alternative approach for treating brain tumor, which can retard glioma growth and prolong animal survival times from orthotopic brain tumor models.

## 1. Introduction

Photothermal therapy (PTT) is one of the least invasive therapeutic modalities for cancer treatment with minimum toxicity. It mainly uses light, usually in the near-infrared (NIR) range for maximum tissue penetration, to directly kill cancer cells while converting light energy into heat. The PTT occurs at higher temperatures than the basal body temperature, usually by achieving a coagulation-threshold temperature above 50 °C, although tumor cell-damaging starts at around 41 °C [1]. The traditional application of PTT does not involve administration of any exogenous photo-absorbing compounds till the use of indocyanine green (ICG) for chromophore-enhanced PTT [2]. Recently, many nanomaterials have been employed as thermal-enhancing agents or a photothermal agents (PAs), to increase the efficiency as well as the targeting ability of PTT [3]. One of major concerns of PTT is the drainage of generated heat into the tumor vicinity, which could lead to unintended damage to neighboring non-tumor cells [4]. This difficulty could be overcome by using a PA for locally induced heat generation upon NIR light irradiation, where selective cancer cell killing occurs only after the intracellular uptake of the PA [5]. Other than ICG, many inorganic nanomaterials such as magnetic nanoparticles, Au nanorods and carbon-based nanomaterials are good PAs [6]. 

Being a form of phototherapy other than PTT, photodynamic therapy (PDT) also involves the use of light, but it uses a photosensitizer (PS) in conjunction with molecular oxygen to elicit death of cancer cells. After activation by light of a specific wavelength, a PS can generate free radicals and/or reactive oxygen species (ROS) from endogenous molecular oxygen to induce therapeutic cytotoxicity toward cancer cells via either cell apoptosis or necrosis [7]. The PDT offers significant advantages for cancer treatment, with greatly reduced long-term morbidity and minimal normal tissue toxicity. The effectiveness of this treatment strongly depends on the type of PS used, its concentration, along with duration of irradiation time and tumor cell oxygen level [8]. The selected PS may offer a photodynamic effect simultaneously with a photothermal effect when exposed to NIR laser irradiation for concurrent PTT/PDT. 

Integrating molecular imaging with therapies in image-guided diagnosis or theranostics has attracted increasing interest recently. The imaging methods include NIR fluorescence imaging, magnetic resonance imaging (MRI), positron emission tomography (PET), ultrasonic imaging and X-ray computed tomography (X-CT) [9]. The NIR fluorescence imaging technique is used for real-time observation under in vivo biological condition. Several NIR fluorescence nanomaterials are commercially available as nanotheranostic agents, among which NIR dyes such as ICG, IR-780, IR-783 and IR-820 are the most extensively used, not only for NIR fluorescence imaging but also for PTT/PDT [6,9,10,11]. The ICG is the only chromophore that is approved by the U.S. Food and Drug Administration (FDA) for clinical imaging and diagnosis and has been used extensively to test liver function as well as in surgical navigation and ophthalmic angiography [12]. Nonetheless, IR-780, a heptamethine dye, was found to be a more powerful and stable nanotheranostic agent than ICG, exhibiting several distinct advantages [12,13]. The hydrophobic nature of IR-780 enables its encapsulation into the bilayer of liposomes for better encapsulation efficiency than ICG-based liposomes [14]. Furthermore, the singlet oxygen (^1^O_2_) quantum yield, photostability and fluorescence intensity of IR-780 are higher than those of ICG. For IR-780, the ^1^O_2_ quantum yield could reach 0.127 vs. 0.002 for ICG [15].

Convection-enhanced delivery (CED) can increase the transport of drug in brain tumors by promoting fluid flow throughout the tumor with a locally applied pressure difference, during which convective diffusion becomes the dominant mechanism of mass transport [16]. This method was first used by a research group at the National Institute of Health in early 1990s [17]. For drug delivery to the brain, CED addresses one of the most difficult challenges faced, namely the blood–brain barrier (BBB), which presents difficulty for delivering drug above a therapeutic concentration window to the brain tumor without demanding administration of toxic quantities of drugs [18]. Another advantage offered by this technique for drug delivery to the brain is the increased tumor selectivity offered by CED for treating glioma. The principle behind CED follows Darcy’s law, where the bulk flow velocity is directly proportional to the pressure gradient. In contrary to conventional drug delivery by diffusive flow based on a concentration gradient, CED requires relatively lesser amounts of drug to achieve similar therapeutic levels [19]. Clinical application of CED involves one or more catheters placed stereotactically through a burr hole into the interstitial spaces of the brain using image guidance. An infusion pump is connected to the catheter(s), which induces a pressure gradient and drives the flow. The drug is directly infused into the extracellular space of the brain, while displacing the extracellular fluid [20]. The convective transport is achieved by interstitial pathways in the brain, which, unlike diffusive transport that occurs during intravenous (IV) drug administration, is driven by the fluid flow velocity and is independent of the size of the molecule to be transported. Indeed, the amount of drug that could be delivered through CED may be 1000- to 10,000-fold high than intravenous (IV) delivery [21].

Although CED of chemotherapeutic drugs to the brain has been reported in glioblastoma treatment, few studies have explored its application for PTT/PDT. On the other hand, as CED will be suitable for delivery of a nanotheranostic agent to the brain for PTT/PDT with unobstructed penetration of laser light through the created burr hole during CED, the stability of a PS during repeated laser irradiation poses another hurdle for clinical application. A prolonged photostability of IR-780 is therefore imperative to meet the need of successful PTT/PDT. Toward this end, we aim to prepare IR-780-loaded liposomes (ILs) and protect hydrophobic IR-780 in the lipid bilayer of liposomes. The ILs after CED will lead to rapid and massive uptake by U87MG human glioblastoma cells surrounding the injection site to improve the outcomes of PTT/PDT with consecutive NIR laser irradiation. The physico-chemical properties as well as the biological responses of liposomal IR-780 were characterized in vitro, followed by concurrent PTT/PDT of intracranial human glioma xenografts in nude mice by CED of ILs in combination with three successive NIR laser treatments.

## 2. Materials and Methods

### 2.1. Materials

Cholesterol (CH), didodecyldimethylammonium bromide (DDAB) and IR-780 were purchased from Sigma-Aldrich (St. Louis, MO, USA). 1,2-Distearoyl-sn-glycero-3-phosphocholine (DSPC) was purchased from Avanti Polar Lipids Inc. (Alabaster, AL, USA). N-(Carbonyl-methoxypolyethylenglycol 2000)-1,2-dipalmitoyl-sn-glycero-3-phosphoethanolamine sodium salt (DSPE-PEG2000) was purchased from NOF Co. (Tokyo, Japan). Cell culture reagents were purchased from Life Technologies (Carlsbad, CA, USA). Female BALB/c nude mice weighing approximately 15–20 g (4–6 weeks old) were procured from the National Laboratory Animal Center (Taipei, Taiwan). 

### 2.2. Preparation of IR-780-Loaded Liposomes (ILs)

The IR-780-loaded liposomes (ILs) were prepared using the thin film hydration method with some modification [22,23]. In brief, lipids (DSPC, CH, DDAB and DSPE-PEG2000) in molar 64/30/4/2 were taken in a round bottom flask filled with chloroform/methanol (2:1, *v*/*v*) to prepare a 10 mM lipid solution. IR-780 dissolved in methanol was added to this mixture to reach a final weight ratio of IR-780:lipids = 3:100. The resultant solution was dried in a rotary evaporator (EYELA N-1200AVF, Tokyo, Japan) to form a thin lipid film after removing the organic solvents, followed by vacuum drying overnight to remove residual solvents. The dried lipid film was then hydrated with 10 mL phosphate buffer saline (PBS) at 55 °C in a water bath for 30 min, after which the solution was sonicated using a probe type sonicator (Q700, Qsonica, Newtown, CT, USA) for 15 min (5 s/5 s on/off pulse cycle, amplitude = 5) followed by a bath type sonicator for 30 min (30 s/5 s on/off pulse cycle, amplitude = 30). The resulting ILs solution was processed with a commercial temperature-controlled barrel extruder (Lipex^®^ Extruder, Transferra Nanosciences, Burnaby, CA, USA) for 10 cycles at 55 °C using double-stacked polycarbonate membranes with 0.2 μm pore size. Subsequently, free IR-780 was removed by dialysis (MWCO = 12–14 KDa) overnight in phosphate buffered saline (PBS) at 4 °C. The encapsulation efficiency (EE) and loading efficiency of IR-780 in ILs was calculated using the following equations [6]:

(1) $$EE\;\left(\%\right)=\frac{Weight\;of\;encapsulated\;IR-780\;}{Weight\;of\;IR-780\;initially\;added}\times 100$$

(2) $$LE\;\left(\%\right)=\frac{Weight\;of\;encapsulated\;IR-780\;}{Weight\;of\;liposomes}\times 100$$

### 2.3. Characteristic of IR-780-Loaded Liposomes (ILs)

The particle size distribution and zeta potential of ILs were determined by dynamic light scattering (DLS) using a Zetasizer Nano ZS (Malvern Panalytical, Malvern, UK) at 25 °C and at a scattering angle of 173° in auto mode. Nanoparticle tracking analysis (NTA) was used to confirm the size distribution of ILs using NanoSight LM10 (Malvern Panalytical, Malvern, UK) equipped with a 405 nm laser. The stability of ILs was measured by statically incubating a 2 mg/mL ILs solution prepared in 95% PBS/5% fetal bovine serum (FBS) at 37 °C. At different time points, samples were removed and analyzed by NTA at room temperature. The samples were measured for 60 s with manual gain and shutter adjustments. The photostability was studied using an ultraviolet–visible (UV–Vis) spectrophotometer (Genesys 150, Thermo Fisher Scientific, Waltham, MA, USA) to monitor the change of solution absorbance of a free IR-780 or ILs (2 μg/mL IR-780) solution prepared in PBS after continuous exposure to day light at 25 °C. For Fourier transform infrared spectroscopy (FTIR) analysis, freeze dried ILs and blank liposomes (without IR-780) were combined with KBr, compressed to form a pellet and analyzed with a Bruker Tensor II FTIR spectrometer (Billerica, MA, USA) from 400 to 4000 cm^−1^ with a 4 cm^−1^ resolution at 2.5 mm/s. 

### 2.4. Photothermal and Photodynamic Effects

The photothermal and photodynamic effects of free IR-780 and ILs were studied separately. For photothermal experiments, aqueous solutions of free IR-780, ILs and PBS in Eppendorf tubes (0.5 mL) were irradiated with 808 nm NIR laser for 5 min and temperature change was monitored using an infrared thermal camera (InfReC Thermo GEAR G100EX, Tokyo, Japan). The concentration of IR-780 in ILs was the same as free IR-780 in all studies. In one study, the IR-780 concentrations were varied from 10 to 50 µg/mL with fixed NIR laser intensity at 1.5 W/cm^2^. In the other study, the IR-780 concentration was fixed (30 µg/mL), while the NIR laser intensity was varied from 1 to 2 W/cm^2^. The photothermal response of free IR-780 and ILs was also studied with repeated on/off laser irradiation cycles. A 0.5 mL solution of free IR-780 or ILs in PBS (40 µg/mL IR-780) was irradiated with three successive NIR laser cycles (1 W/cm^2^, 3 min on/15 min off). The change in temperature was monitored with an infrared thermal camera, and gross change of solution color was captured with a digital camera.

The photodynamic effects of free IR-780 and ILs were determined from reactive oxygen species (ROS) generation using 1,3-diphenyl isobenzofuran (DPBF) as a chemical probe [24]. Ten microliters of DPBF (2 mg/mL in acetonitrile) was mixed with 1 mL of free IR-780 or ILs (5 µg/mL IR-780 in acetonitrile) and irradiated with 808 nm NIR laser (1 W/cm^2^). At predetermined time points, the solution absorbance was immediately measured at 410 nm using an UV–Vis spectrophotometer. A blank prepared in acetonitrile was irradiated similarly with NIR laser and used as a control. 

Intracellular ROS detection was measured with a cell permeable non-fluorescent probe, 2′,7′-dichlorofluorescin diacetate (DCFH2-DA) [25]. Briefly, U87MG cells (1 × 10^4^) were seeded in a 24-well cell culture plate and cultured overnight. After washing with PBS, the cells were treated with free IR-780 or ILs (2 µg/mL IR-780 in cell culture medium) for 2 h. After washing with PBS, the cells were further incubated with DCFH2-DA (20 µM) for 60 min at 37 °C. After washing with PBS and replenishing with cell culture medium, the cells were treated with NIR laser for 3 min at 1.5 W/cm^2^. Control was PBS treatment without NIR laser exposure. The fluorescence due to ROS generation was detected by observation under an inverted fluorescence microscope (Olympus IX-71, Tokyo, Japan). The quantitative intercellular ROS was determined from flow cytometry using DCFH2-DA [26]. After seeding 3 × 10^5^ cells/well U87MG in a 6-well cell culture plate overnight, the cells were washed with PBS and cultured in cell culture medium containing free IR-780 or ILs (2 µg/mL IR-780) for 2 h at 37 °C. The cells were washed again with PBS and incubated with DCFH2-DA (20 μM) for 60 min. After further washing with PBS, the cells were treated with NIR laser for 3 min (1.5 W/cm^2^) in cell culture medium. The fluorescence (ROS) was detected by Thermo Attune NxT flow cytometer (Waltham, MA, USA) at 488 nm excitation and 530 nm emission wavelengths.

Western immunoblot was conducted to understand the anticancer molecular mechanism. The expression of heat shock protein 70 (HSP70) was determined in U87MG cells after exposing to free IR-780 or ILs with or without NIR laser irradiation (1.5 W/cm^2^) for 3 min [27]. Briefly, 1 × 10^6^ cells were seeded in T-75 flask and culture overnight. After incubating with free IR-780 or ILs (5 µg/mL IR-780) for 2 h, the cells were trypsinized and treated with NIR laser in an Eppendorf tube. After incubation for another 10 h, cells were washed and lysed by radioimmunoprecipitation assay (RIPA) buffer containing protease inhibitors. The supernatant was recovered, and the protein concentration was measured with BCA protein assays after centrifugation to remove cell debris. The protein was heat-denatured for 10 min in a sample buffer at 95 °C, followed by separating aliquots of lysate by polyacrylamide sodium dodecyl sulfate gel electrophoresis at 50 V for 30 min (~25 μg total protein/lane) and at 110 V for 2 h. The gels were transferred to a polyvinylidene fluoride membrane, blocked for non-specific binding with 5% fat-free milk for 1 h and treated with primary antibodies for HSP70 (ab231637, Abcam, Cambridge, UK) and β-actin (13E5, Cell Signaling Technology, Danvers, MA, USA) overnight at 4 °C. After washing with Tris-buffered saline and Tween 20 (TBST) 3 times, secondary antibody (anti-rabbit IgG-HRP, 1:2000) was added, followed by ECL Western blotting substrate for color development. The band densitometry analysis was carried out using ImageJ software (National Institute of Health Bethesda, MD, USA) for relative protein expression after detection with a MultiGel-21 gel image system (Top Bio Co., Taipei, Taiwan). 

### 2.5. Intracellular Uptake and Cytotoxicity

For in vitro experiments, U87MG human primary glioblastoma cells (ATCC HTB1) were obtained from the American Type Culture Collection (Manassas, VA, USA). To study the in vitro cell cytotoxic effect of free IR-780 and ILs with or without NIR laser irradiation, U87MG cells were seeded in a 96-well cell culture plate at 5 × 10^3^ cell/well and cultured overnight in cell culture medium (90% high glucose Dulbecco’s modified Eagle’s medium (DMEM) and 10% FBS) in a humidified CO_2_ incubator at 37 °C under 5% CO_2_. The cell culture medium was replaced with cell culture medium containing different concentrations of IR-780 or ILs and incubated at 37 °C for 12 h in a humidified CO_2_ incubator. Each well was treated with NIR laser for 4 min at 1.5 W/cm^2^. The cell viability was determined from (3(4,5-dimethylthiazol-2-yl)-2,5-diphenyltetrazolium bromide) (MTT) assays by measuring the solution absorbance at 540 nm using a microplate reader [28]. 

The flow cytometry study for apoptotic and necrotic cell distribution assays was performed with fluorescein isothiocyanate-labeled Annexin V (FITC–Annexin V) and propidium iodide (PI). At a cell seeding density of 4 × 10^5^ cells/well, U87MG was used in a 6-well cell culture plate, and cell culture was carried out overnight in a humidified CO_2_ incubator at 37 °C under 5% CO_2_. The cells were washed with PBS and cultured in fresh cell culture medium containing IR-780 or ILs (4 µg/mL IR-780) for 6 h. After washing in PBS, cells were trypsinized and collected in a glass tube (500 µL in cell culture medium). The laser treatment groups received NIR laser treatments for 5 min at 1.5 W/cm^2^. The cell suspension was reacted for 30 min with FITC–Annexin V followed by PI for flow cytometry analysis (Attune NxT flow cytometer) after adding 500 μL fresh cell culture medium.

The intracellular uptake of free IR-780 and ILs was studied with U87MG cells. For this, 4 × 10^4^ cells were seeded on 15 mm coverslips placed in a 24-well cell culture plate and cultured overnight with cell culture medium in a humidified 5% CO_2_ incubator at 37 °C. The cells were further washed with PBS and cultured in cell culture medium containing free IR-780 or ILs (2 µg/mL IR-780) for predetermined times. The lysosomes were labeled with LysoTracker Green DND-26 (1 μM) for 60 min at 37 °C after washing cells with PBS. Further, the labeled cells were washed with PBS, fixed with 4% paraformaldehyde and treated with Triton X-100 (0.1% in PBS). In the final step, the labeled cells were counterstained for cell nucleus with Hoechst 33342 (1 μg/ mL) for 15 min. For confocal laser scanning microscopy analysis, the coverslips were carefully removed and fixed to a glass plate using mounting medium. Cellular internalization was measured by an inverted confocal microscope LSM 510 Meta (Zeiss, Oberkochen, Germany), with excitation/emission wavelengths of 350 nm/451 nm, 504 nm/511 nm and 684/784 nm, for blue, green and red fluorescence, respectively. The red fluorescence was used for the identification for IR-780 or ILs, green for lysosomal identification and blue for cell nuclei [29]. 

### 2.6. Intracranial Xenograft Tumor Model 

All animal experiments protocols were approved by the Chang Gung University’s Institutional Animal Care and Use Committee (IACUC Approval No.: CGU105-034). To establish a human glioma xenograft model in nude mice, U87MG human glioblastoma cell lines genetically engineered to express firefly luciferase genes were implanted intracranially in BALB/c nude mice [30]. Prior to implantation, the animals were anesthetized with 3% isoflurane gas, and a sagittal incision was made in the skin overlying the calvarium, followed by creating a burr hole on right side of the brain at the center region between the bregma and lambda using a 26-gauge needle. Three microliters of U87MG cell suspensions (1 × 10^5^ cells/μL in cell culture medium) was injected using a 10 μL Hamilton syringe 3 mm below the brain surface, before extracting the needle in 100 s. The tumor development was confirmed by bioluminescence imaging (BLI) using a non-invasive in vivo imaging system (IVIS) (Xenogen IVIS-200, Caliper Life Sciences, Waltham, MA, USA). The timeline for establishing the intracranial xenograft tumor model, convention-enhanced delivery (CED) and assessment of anti-tumor efficacy is shown in Figure S1.

### 2.7. Convection-Enhanced Delivery (CED) 

The CED infusion cannulas were fabricated by taking a 20 cm Polymicro capillary tube (inner diameter = ~98 µm; outer diameter = ~240 µm) at one end. For infusion, a 0.3 mm stepped-tip was created with a 2 cm Polymicro capillary tube (inner diameter = ~323 µm, outer diameter = ~430 µm) and fixed with resins and molded with Super Flangeless (P-259X) rings. The other end was connected to a connecter (luer Adapter ¼-28 female to female luer), which could be fitted into a 100 µL Hamilton syringe. The U87MG tumor-bearing mice were randomly divided into three groups, namely PBS + laser, free IR-780 + laser and ILs + laser (*n* = 4, each group), on day 6 post-implantation of U87MG cells. The tumor-bearing mice were anesthetized with liquid isoflurane (Zoletil/Rompum = 4/1, diluted with saline 1:1) and held in fixed position using a stereotactic holder, while a sagittal incision was made in the skin overlying the calvarium. The animal received CED of PBS, free IR-780 or ILs (0.8 µg/µL IR-780) from a Hamilton syringe connected to CED infusion cannulas and mounted in a two-channel laboratory syringe pump (Figure S2). A total volume of 10 µL sample was infused at a 0.5 μL/min flow rate, and the CED infusion cannulas were withdrawn 2 min post-delivery. For laser light treatment, the light was delivered through the same burr hole created during CED.

### 2.8. Bio-Distribution and In Vivo Fluorescence Imaging 

The bio-distribution and in vivo degradation of ILs was performed by administrating 10 μL of ILs (0.5 μg/μL IR-780) or PBS (control) via CED on day 6 post-implantation of U87MG cells. The brain plus other major organs from a mouse were explanted after euthanasia on day 6, 8, 10 and 12, for ex vivo NIR fluorescence imaging using Xenogen IVIS-200 at excitation/emission wavelengths of 745 nm/850 nm.

### 2.9. In Vivo Photothermal Effects 

On day 7 post-implantation of U87MG cells (1 day post-CED), mice in all three groups received the first NIR laser irradiation (808 nm) for 5 min on top of the closed sagittal incision in their head. The laser power used was at 1 W/cm^2^, to maintain a temperature below 55 °C in the brain. The real-time temperature profiles in tumor-bearing mice during laser irradiation were monitored using an infrared thermal camera (InfReC Thermo GEAR G100EX, Tokyo, Japan). The distance between the NIR laser head and the tumor was 30 cm. The second and third laser treatment were performed on day 9 and day 11, respectively, following the same protocol as the first treatment. 

### 2.10. In Vivo Anti-Tumor Efficacy 

The in vivo antitumor efficiency was monitored from bioluminescence signal intensity from BLI. On day 5, 7, 9, 12, 14 and 16 post-implantation of tumor cells, 100 µL of luciferase solution (15 mg/mL) was intraperitoneally injected into mice for BLI within 10 min. By using the Living Image^®^ 4.0 program (PerkinElmer, Waltham, MA, USA), the BLI intensity was determined as baseline on day 5 (i.e., before treatment) and also at each time point after treatment by calculating the total BLI peak intensity within the standardized region of interest (ROI) in the tumor. The BLI signal intensities at each time point were normalized with the baseline value on day 5. All mice were observed regularly and euthanized after they had reached moribund condition due to body weight loss of more than 25% from the initial value, or showing back hunk, hemiparesis and seizures. In order to determine systemic toxicity, a blood sample was collected in a blood collection tube after sacrificing the animal and subjected to basic hematological as well as biochemical analysis of major organ function.

### 2.11. Magnetic Resonance Imaging (MRI) and Positron Emission Tomography/Computed Tomography (PET/CT) Study 

For visualization of tumor in mice brain, magnetic resonance imaging (MRI) was carried out using a Siemens 3.0 Tesla scanner (Magnetom Trio, Siemens, Munich, Germany) equipped with a wrist coil to achieve the relaxation of MRI. During the MRI on day 7 (before laser treatments), 13 and 16 (after laser treatments), mice were anesthetized under isoflurane (2.5%) inhalant. For the T1* series, the image parameters used were as follows: gradient echo, TR/TE: 230/3.81 ms; flip angle: 70; slice thickness: 0.5 mm. The tumor volume in mouse brain was determined from the ROI by multiplying the summed up areas traced on each coronal T2-weight image with the slice thickness [31]. For the positron emission tomography/computed tomography (PET/CT) study, a NanoScan PET (PET122S, Mediso, Budapest, Hungary) was used. Mice were anesthetized with 2% isoflurane on day 5 (before laser treatment), as well as on day 12 (after laser treatment), and intravenously administrated with two different tumor uptake markers, namely Ga68 RGD (0.3 ± 10% mCi) (1 Curie (Ci) = 3.7 × 10^10^ decays/sec) or Ga68 FAPI (0.4 ± 10% mCi). PET imaging parameters used were as follows: 360 projection; 50 kVp 980 μA; 170 ms exposure time; 1:4 binning; helical acquisition; pitch 1; voxel size 250 × 250 × 250 μm. Using the PMOD 4.004 software, images were analyzed, and tumor uptake was quantified from the maximum standardized uptake value (SUV_max_).

### 2.12. Histological Analysis

The brain and major organs were carefully removed and preserved in formaldehyde after sacrificing the animal. To conduct hematoxylin and eosin (H&E) staining of tumor tissue and explanted organs, the sample was treated with phosphate buffered formalin, followed by paraffin embedding for sectioning into 5-μm thickness. For immunohistochemical (IHC) staining, the cell proliferation marker Ki-67 and cell apoptosis marker cleaved caspase 3 (CC3) in tumor tissue was detected with primary antibody, followed by the UltraVision™ Quanto Detection System HRP DAB (Thermal Fisher Scientific, Waltham, MA, USA), and counterstained with hematoxylin. The primary antibody used was anti-Ki-67 (1:200) and CC3 (1:100) monoclonal antibodies from rabbit. The slides were analyzed with a TissueFAXS inverted bright field scanning system (TissueGnostics GmbH, Vienna, Australia), and immunoreactivity was quantified using the PAX-it image analysis software within a ROI of 280 × 200 µm dimensions (*n* = 3). To evaluate safety of the treatment, the major organs from sacrificed mice were examined after H&E staining.

### 2.13. Statistical Analysis

All data were reported as mean ± standard deviation (SD). The one-way analysis of variance (ANOVA) analysis with Tukey honestly significant difference (HSD) test was used to compare subgroups and statistical significance was declared at *p* < 0.05.

## 3. Results and Discussion

### 3.1. Characterization of ILs

**Figure 1:**
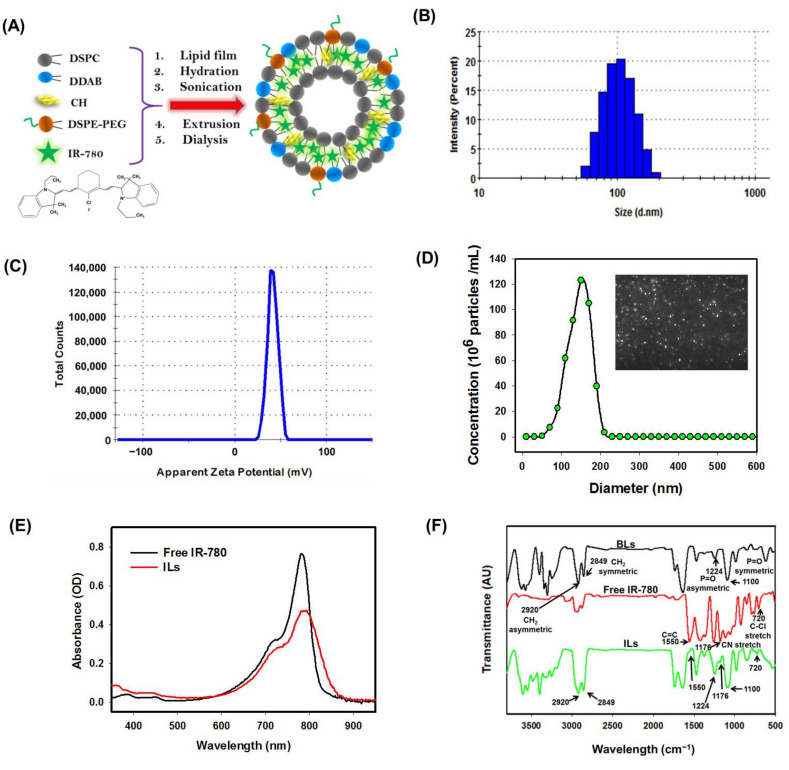
Preparation and characterization of IR-780-loaded liposomes (ILs). (**A**) The schematic diagram showing the structure of ILs. (**B**) The distribution curves of particle size from dynamic light scattering (DLS). (**C**) The distribution curves of zeta potential. (**D**) The particle size distribution from nanoparticle tracking analysis (NTA) with the insert showing light scattering particles taken from the screenshot of original video file. (**E**) The ultraviolet–visible (UV/Vis) absorption spectra of free IR-780 (4.5 µg/mL) and ILs (2.7 µg/mL IR-780) in PBS. (**F**) The Fourier-transform infrared (FTIR) spectra of blank liposomes (BLs) (liposomes without IR-780), free IR-780 and ILs.

We prepared ILs from DSPC, CH, DDAB and DSPE-PEG2000 by the thin film hydration method for entrapment of hydrophobic IR-780 within the lipid bilayer (Figure 1A). The CH plays a role in stabilizing ILs [32]. By incorporating DDAB, cationic ILs is expected to be accumulated around cancer cells by binding to negatively charged cell membrane as well as fusion with cell membrane for facilitated intracellular uptake. Previously, the PEGylated positively charged liposome was shown to increase the survival time of tumor-bearing rats after CED of liposomal carboplatin [33]. From the distribution curve of hydrodynamic diameter measured by DLS (Figure 1B) and zeta potential (Figure 1C), the average size and zeta potential was 136.6 ± 5.5 nm and 33.3 ± 5.1 mV, with a polydispersity index (PDI) of 0.21 ± 0.03 (*n* = 3). Consistent with DLS, the diameter of ILs was 145 ± 26 nm from NTA, shown as fairly monodispersed light scattering particles from the screenshot image due to its cationic nature (Figure 1D).

The UV–Vis absorption spectrum of free IR-780 showed a strong absorption peak at 780 nm, which shifted slightly to ~790 nm for ILs, indicating successful encapsulation of IR-780 within ILs (Figure 1E). The strong absorption peak shown by ILs in the NIR region supports its use as a PTT/PDT agent with NIR laser light [34]. The EE and LE of IR-780 in ILs were calculated to be 82.0 ± 3.4% and 1.10 ± 0.14% (*n* = 3), respectively, from a linear calibration curve constructed at 780 nm. From FTIR analysis, blank liposomes (without IR-780) revealed the symmetric and asymmetric stretch modes of phosphate group (P=O) at 1100 cm^−1^ and 1224 cm^−1^ as well as CH_2_ symmetric and asymmetric stretch modes at 2849 cm^−1^ and 2920 cm^−1^ from the major lipid component DSPC (Figure 1F) [6]. The free IR-780 revealed the aliphatic chloro compounds’ C–Cl stretch at 720 cm^−1^, CN stretch at 1170 cm^−1^ and aromatic ring stretch of C=C at 1550 cm^−1^. The encapsulation of IR-780 resulted in additional peaks that were assigned to IR-780 in the FTIR spectrum of ILs as expected [35]. 

**Figure 2:**
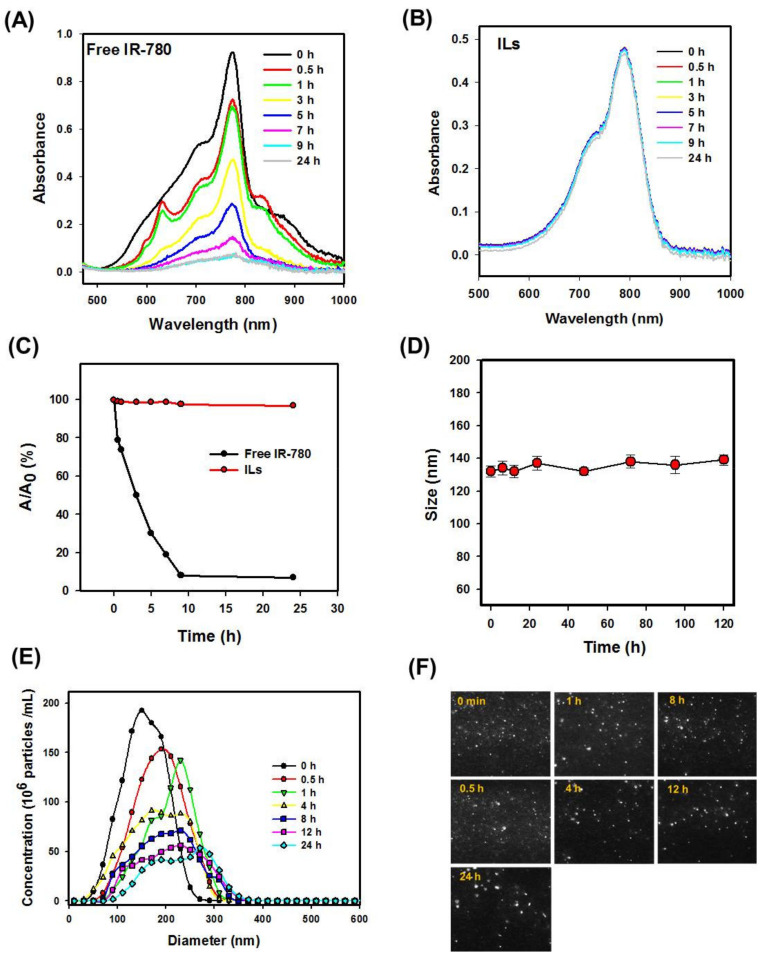
The stability of IR-780-loaded liposomes (ILs). The photostability of free IR-780 and ILs from ultraviolet–visible (UV/Vis) absorption spectra of free IR-780 (**A**) and ILs (**B**) in PBS under natural daylight exposure at room temperature was compared from normalized absorbance (A/A_0_) at different time points (**C**). The colloidal stability of ILs was determined from the change in average particle size in phosphate buffered saline (PBS) with dynamic light scattering (DLS) (**D**), the change in particle size distribution in 5% fetal bovine (FBS)/95% PBS with nanoparticle tracking analysis (NTA) (**E**) and the screen shots of light scattering particles from original NTA video files (**F**).

To study the photostability of IR-780, free IR-780 and ILs in PBS were exposed to natural daylight at room temperature, and the absorption spectra were recorded with an UV–Vis spectrophotometer. As shown in Figure 2A, the maximum solution absorbance of free IR-780 decreased continuously with time to nearly zero after 9 h, indicating free IR-780 is unstable under light exposure. On the other hand, the maximal solution absorbance of ILs remained almost constant within 9 h, followed by a minimum decrease in 24 h (Figure 2B). For better comparison of photostability, the maximum absorbance (A) at different time points was normalized with its initial value at time 0 (A_0_). As shown in Figure 2C, no noticeable difference in A/A_0_ was found for ILs till 24 h, in contrast to free IR-780, which showed fast degradation with less than 10% initial absorbance value after 9 h. The enhancement of IR-780 stability could be due to entrapment of the PS within the lipid bilayer of ILs, which is consistent with other reports showing improved stability of entrapped IR-780 or nanoparticle-conjugated IR-780 [12,13,34,36]. 

The colloidal stability of ILs was studied from the size (hydrodynamic diameter) change of ILs after incubating in PBS for different times. As shown in Figure 2D, the average particle size was within 132 to 140 nm, with no significant difference found up to 120 h. The stability of ILs in 5% serum/95% PBS was determined from NTA up to 24 h, shown as concentrations of ILs vs. particle diameter in Figure 2E. No noticeable difference in the size of the dispersing light was observed at any time from the screenshot images in Figure 2F. The particle size shifted to a higher value with diminished particle concentration at longer incubation time, indicating time-dependent destruction of ILs. Many factors can influence the stability of liposomes, and the change in the concentration of ILs with time may be due to the destruction or aggregation of liposomes, leading to increased particle size and decreased particle concentration from NTA counts [37]. Unlike drug delivery by IV injection where long circulation time may be required before reaching the tumor site, CED could be completed within a much shorter period of time. Thus, ILs should be suitable for CED, considering their stability in vitro [38].

### 3.2. In Vitro Photothermal and Photodynamic Study 

**Figure 3:**
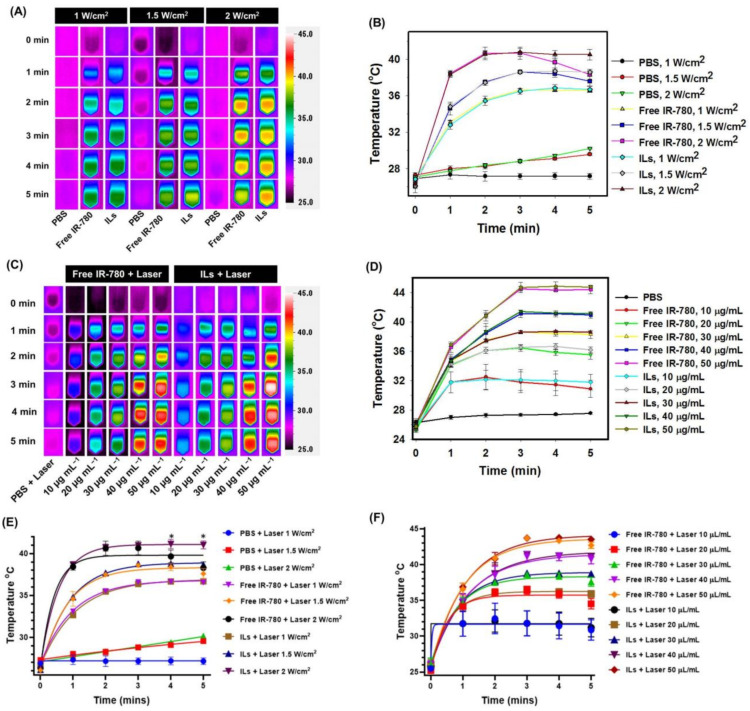
The in vitro photothermal effects with near infrared (NIR) laser irradiation. (**A**) The thermal images and (**B**) the peak temperature profiles of phosphate buffered saline (PBS), free IR-780 and IR-780-loaded liposomes (ILs) (30 μg/mL IR-780) after irradiating with 808 nm NIR laser at 1, 1.5 or 2 W/cm^2^. (**C**) The thermal images and (**D**) the peak temperature profiles of PBS, free IR-780 and ILs (10, 20, 30, 40 or 50 μg/mL IR-780) after irradiating with 808 nm NIR laser at 1.5 W/cm^2^. (**E**,**F**). The kinetics of peak temperature profiles are plotted using one-phase association fits. There is significant difference between Free IR-780 + laser and ILs + laser on 4 and 5 min at 2 W/cm^2^ (* *p* < 0.05).

The in vitro photothermal effects of free IR-780 and ILs were studied after irradiating with NIR laser with real-time change in solution temperature monitored with an infrared (IR) thermal camera. The maximum temperature acquired from the IR camera image was used to plot the real-time temperature profile. When PBS was exposed to NIR laser at 1 W/cm^2^ intensity for 5 min, no substantial change in temperature was observed, although a minor temperature change from 27 °C to 30 °C was noted at 1.5 and 2 W/cm^2^ (450 and 600 J/cm^2^) (Figure 3A,B). In contrast, when free IR-780 or ILs of the same IR-780 concentration was exposed to 1 W/cm^2^ laser irradiation, the temperature reached 36 °C in 5 min. At 1.5 and 2 W/cm^2^ intensity, the temperature rose to 38 °C and 41 °C in 3 min for free IR-780 and ILs, with no significant difference found between them. Nonetheless, a distinctive temperature profile was found between free IR-780 and ILs after 3 min, with only free IR-780 showing a drop in temperature to 37 °C (at 1.5 W/cm^2^) or 38 °C (at 2 W/cm^2^) in 5 min. A temperature drop shown only by free IR-780 within 3 to 5 min, but not by ILs, underlines improved photostability of IR-780 in ILs with continuous decomposition of free IR-780 under NIR laser irradiation. This leads to retarded photothermal response with time, shown from a temperature drop at a longer time when the rate of heat dissipation is higher than the rate of heat generation. This difference is consistent with difference of decomposition rate between ILs and free IR-780 under daylight displayed in Figure 2C. Overall, ILs could be suggested as a preferred source for PTT over free IR-780 when exposed to NIR laser light. 

The concentration-dependent photothermal effects at a fixed NIR laser intensity (1.5 W/cm^2^) was examined next for free IR-780 and ILs with varying IR-780 concentrations. As shown in Figure 3C,D, the maximum temperature could be maintained at 38, 40 or 44 °C after 3 min at 30, 40 or 50 µg/mL, without significant difference found between free IR-780 and ILs. Nonetheless, at 10 and 20 μg/mL concentrations, free IR-780 showed a temperature increase to 32 and 35 °C, respectively, for the first 3 min, and a slight decrease to 31 and 34 °C, respectively, in 5 min. In contrast, although the temperature of ILs increased similarly to 32 and 35 °C in 3 min, it remained at this value thereafter till 5 min. From the in vitro photothermal study, we conclude that exposing 30 μg/mL of free IR-780 to 1.5 or 2 W/cm^2^ NIR laser can result in a temperature rise suitable for PTT in 3 min. However, it fails to maintain this threshold PTT temperature for a longer duration. Only at a lower laser intensity (1 W/cm^2^) and higher PS concentrations (30, 40 or 50 µg/mL) could free IR-780 show comparable photothermal efficiency with ILs. The kinetics of peak temperature profiles are plotted using one-phase association fits and are shown in Figure 3E,F.

**Figure 4:**
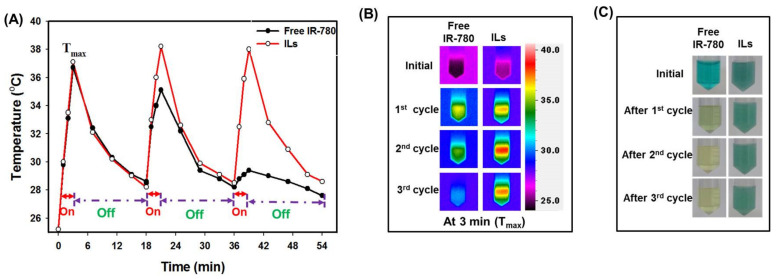
The photothermal stability of IR-780-loaded liposomes (ILs) and free IR-780. (**A**) The peak temperature profiles of free IR-780 and ILs (40 μg/mL IR-780) during three on/off laser cycles. The near infrared (NIR) laser was used at 1 W/cm^2^ with 3 min on and 15 min off. (**B**) The corresponding thermal images at T_max_ (3 min) for free IR-780 and ILs during each laser irradiation cycle. (**C**) The appearance of free IR-780 and ILs solution at the end of each laser irradiation cycle.

Other than continuous laser exposure, we compared the photostability between IR-780 and ILs after three repeated on/off laser cycles, consisting of 3 min on at 1 W/cm^2^ and 15 min off. As shown from the peak temperature profiles in Figure 4A, free IR-780 and ILs reached a maximum temperature (T_max_) of 37 °C in 3 min and dropped to 29 °C after turning off the laser in the first cycle. In second cycle, free IR-780 reached a T_max_ of 35 °C while ILs reached 38 °C. During the final cycle, free IR-780 only managed to reach a T_max_ of 29 °C, in contrast to 38 °C for ILs. The 3 °C temperature difference between free IR-780 and ILs in the second cycle was due to the decomposition of IR-780 during the first cycle. Nonetheless, the drastic difference in photostability between IR-780 and ILs led to a 9 °C temperature difference in T_max_ during the third cycle, due to vast decomposition of IR-780 during the first two laser irradiation cycles. The ILs managed to reach similar T_max_ within 3 min in all laser cycles due to protection of IR-780 by the lipid bilayer of ILs. The drastic change of thermal images at T_max_ at 3 min (Figure 4B) and the pronounced change of solution color (Figure 4C) between free IR-780 and ILs support changes in photothermal and chemical properties of IR-780 under the influence of NIR laser and endorses the use of ILs for PTT/PDT. Using self-assembled IR-780 containing micelles for PTT, Yuan et al. found the temperature rise significantly reduced after the first laser cycle during repeated laser irradiation (808 nm, 1 W/cm^2^) [39]. In comparison, liposomes loaded with IR-780 are more stable after repeated laser irradiation, as temperature rise could be retained till three cycles, and lipid bilayers were suggested to offer protection of IR-780 against generated singlet oxygen [23]. 

For PDT, the photodynamic effects of IR-780 were studied with DPBF as a chemical probe. The singlet oxygen (^1^O_2_) or reactive oxygen species (ROS) produced from a PS after exposure to NIR laser is responsible for cytotoxicity in PDT [40,41,42]. The DPBF will react with ^1^O_2_ to give endoperoxides by 1,4-cycloaddition, which undergoes decomposition to generate o-dibenzoylbenzene at room temperature [43,44]. Although several other chemical methods could be used to detect ^1^O_2_, the difference in absorption spectra between IR-780 and DPBF as well as the strong absorbance at 410 nm shown only by DPBF but not by its decomposed products from loss of the isobenzofuran π system, make facial detection of singlet oxygen possible [45]. As shown in Figure S3A and B, the decrease of solution absorbance at 410 nm after NIR laser irradiation for ROS generation is different for free IR-780 and ILs. The ability to generate ROS was compared by normalizing the maximum solution absorbance at different times (A) with its initial value at time 0 (A_0_) in Figure S3C. During the first 10 s, the A/A_0_ value was the same for IR-780 and ILs; nonetheless, free IR-780 showed much faster generation of ROS compared to ILs with comparatively smaller A/A_0_ values. The A/A_0_ values for free IR-780 and ILs decreased to 8% and 38%, respectively, 120 s after laser irradiation. This difference may have arisen as IR-780 was protected from NIR light within the lipid bilayer of ILs, consuming less energy and generating less ROS. You et al. also reported a similar behavior with a different PS (ICG) loaded in mesoporous silica-coated copper sulfide nanoparticles [24].

### 3.3. In Vitro Cell Culture Experiments

**Figure 5:**
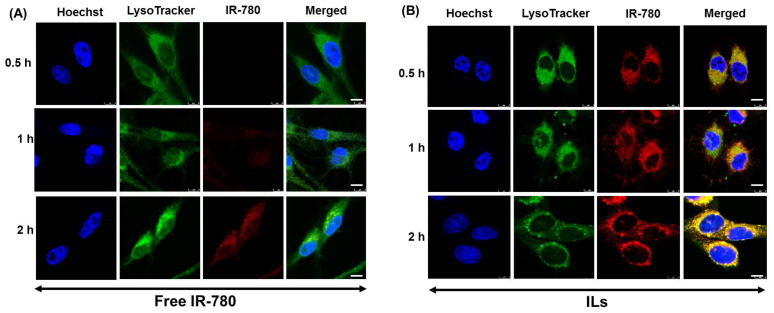
Intracellular uptake studies using confocal laser scanning microscopy. The intracellular uptake of free IR-780 (**A**) and IR-780-loaded liposomes (ILs) (**B**) by U87MG cells using free IR-780 or ILs (2 µg/mL IR-780). Scale bar = 8 μm.

We first studied the intracellular uptake kinetics of free IR-780 and ILs by U87MG using confocal laser scanning microscopy. As shown in Figure 5A, there was no indication of red fluorescence signal after treating cells with free IR-780. The red fluorescence started to show after 1 h and continued to increase after 2 h. However, in case of ILs, strong red fluorescence intensity could be observed in cell cytoplasm as early as 0.5 h, which gradually increased with incubation time (Figure 5B). From merged images, high fluorescence intensity (yellow) corresponding to both IR-780 (red) and LysoTracker (green) was shown only for ILs but not for free IR-780, suggesting efficient uptake of ILs via endocytosis. That IR-780 shows much improved cellular trafficking rate upon entrapment in ILs could be related to the cationic nature of ILs (zeta potential = 33.3 mV), which could promote charge-mediated endocytosis of liposomes through passive accumulation and selective binding of ILs to the tumor cell surface, in contrast to free IR-780 that has negligible positive charge [29,46]. This is consistent with previous reports that cationic liposomes can fuse with anionic endosome membrane by electrostatic interaction [47], and cationic PEGylated liposomes had the most proficient cellular uptake in vitro [48].

**Figure 6:**
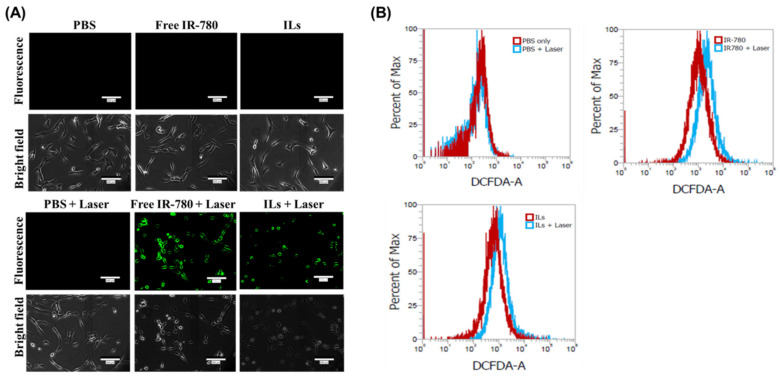
Intracellular ROS production by U87MG cells observed under an inverted fluorescence microscopy (bar = 200 μm) (**A**) and analysis by flow cytometry (**B**). The cells were treated with PBS, IR-780 or IR-780-loaded liposomes (ILs), followed by irradiation with 1.5 W/cm^2^ near infrared (NIR) laser for 3 min.

For production of intracellular ROS, DCFH2-DA was chosen as an ROS probe. The DCFH2-DA can diffuse into U87MG cells and be deacetylated by cellular esterases to produce a non-fluorescent product, which is oxidized by ROS into a highly fluorescent product DCF for detection by fluorescence microscopy. To this end, U87MG cells were treated with free IR-780 or ILs for 2 h and washed to study intracellular uptake. The cells were incubated with DCFH2-DA for 1 h for permeation of DCFH2-DA into cells, followed by laser irradiation for 3 min at 1.5 W/cm^2^. As shown in Figure 6A, no green fluorescence signal could be observed for the PBS (control), free IR-780 and ILs groups without laser irradiation. In contrast, a strong green fluorescence signal was shown only in free IR-780 + laser and ILs + laser groups, indicating intracellular ROS was produced from endocytosed IR-780 after NIR irradiation [25]. Consistent with the ability to produce extracellular ROS, as shown in Figure S3, free IR-780 appeared to display a more intense green fluorescence signal than ILs, suggesting higher intracellular ROS production ability from free IR-780. 

The quantitative evaluation of intracellular ROS production was confirmed from flow cytometry analysis. As shown in Figure 6B, there was no peak shift in the PBS control group even with laser irradiation. In contrast, an obvious shift of peak fluorescence intensity was detected in free IR-780- and IL-treated cells upon laser exposure due to intracellular ROS production. The change of mean fluorescence intensity was 2.3-fold for free IR-780 (from 1375 to 3187) and 1.5-fold for ILs (from 1214 to 1840). These findings suggest that free IR-780 shows stronger ROS production ability than ILs upon NIR laser exposure, albeit with less photostability. 

To understand the potential molecular mechanism of PTT/PDT after laser treatment of U87MG cells, we investigated the expression of a thermal stress-related protein, heat shock protein 70 (HSP70), which is typically overexpressed in most cancer cells or organelles, aiding cancer survival by preserving the protein homeostasis [49,50]. Increased HSP70 expression can also predict anti-tumor response [27,50], although decreased expression is associated with drug resisting cells in chemotherapy [50,51]. As shown in Figure S4, U87MG cells in free IR-780 + laser and ILs + laser groups show elevated expression of HSP70 from Western blotting, due to thermal stress experienced by the cells from the IR-780-induced photothermal response [27]. There was a significant difference in relative HSP70 protein expression by the action of NIR laser for free IR-780 and ILs, which was 1.24 ± 0.06 and 1.63 ± 0.07, respectively. Comparing free IR-780 and ILs upon laser exposure, the less pronounced upregulation of HSP70 protein expression with free IR-780 may have been due to the higher rate of ROS generation, which resulted in reduced thermal stress experienced by U87MG cells. 

**Figure 7:**
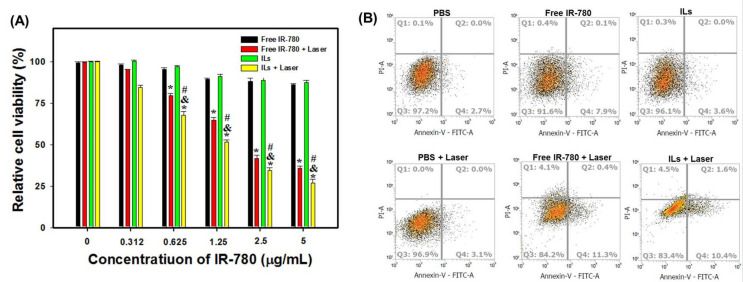
Cell cytotoxicity from MTT assays and flow cytometry. (**A**) In vitro cell cytotoxicity of free IR-780 and IR-780-loaded liposomes (ILs) with or without near infrared (NIR) laser treatment (1 W/cm^2^ for 4 min) from MTT assays. * *p* < 0.05 compared with free IR-780, ^&^ *p* < 0.05 compared with ILs, ^#^ *p* < 0.05 compared with free IR-780 + laser. (**B**) The flow cytometry analysis of apoptotic and necrotic cells with fluorescein isothiocyanate-labeled Annexin V (FITC–Annexin V) and propidium iodide (PI).

After confirming the photothermal and photodynamic effects in vitro, concurrent PTT/PDT was studied by incubating U87MG cells with free IR-780 or ILs of different IR-780 concentrations for 12 h. The cells were irradiated with NIR laser at 1.5 W/cm^2^ for 4 min to induce cytotoxicity, followed by the determination of cell viability by MTT assays. As shown in Figure 7A, free IR-780 or ILs showed negligible cytotoxicity up to 1.25 µg/mL concentration. Under laser exposure to induced PTT/PDT in vitro, free IR-780 or ILs showed drastically reduced cell viability, which was dose-dependent on IR-780 concentration. Most important, ILs exhibited significantly higher cytotoxicity toward U87MG compared to free IR-780, implicating differences in photostability between free and entrapped IR-780 upon continuous laser irradiation, as well as possible differences in their intracellular uptake, which may lead to significant differences in cancer cell killing effect with improved PTT/PDT by using ILs.

The laser-induced cytotoxic due to free IR-780 and ILs (4 µg/mL IR-780) was further studied from flow cytometry analysis of cell apoptosis/necrosis (Figure 7B). To evaluate the percentage of live (Q3), early apoptotic (Q4), late apoptotic (Q2) and necrotic (Q1) cells based on variations in permeability and integrity of cell membranes, we used Annexin V/PI to stain the cells after different treatments in vitro. As shown in Figure 7B, cells in PBS control without NIR laser irradiation gave 97.2% live cells and a 3% apoptosis rate. A negligible change in cell viability (96.9%) was noted from the PBS + laser group. Without laser irradiation, free IR-780 resulted in an 8.0% apoptosis rate with 91.6% live cells. In contrast, ILs treatment showed a 3.6% apoptosis rate and 96.1% live cells. These findings further support the minimum cytotoxicity of ILs observed in Figure 7A, while entrapment in the lipid bilayer of ILs may even enhance the biocompatibility of IR-780. After laser irradiation, cells in free IR-780 showed an increase of necrosis rate, from 0.4% to 4.1%, while the apoptosis rate also increased to 11.7%. Similarly, laser irradiation of ILs also led to a vast increase of necrosis rate, from 0.3% to 4.5% and 12.0% cell apoptosis. Overall, a reduced percentage of viable cells with increased apoptotic and necrotic cells is consistent with the results from MTT assays of cell viability, which is based on mitochondria activity, and supports the use of ILs plus laser irradiation for concurrent PTT/PDT.

The laser fluence rate usually does not exceed 250 mW/cm^2^ in PDT due to extensive photobleaching of the photosensitizer and thermal effects that predominate over the PDT effects at higher rates. However, it is difficult to completely rule out the contribution of PDT in the current study, as PTT and PDT are closely related by intersystem crossing of energy states, through energy dissipation during movement of electrons upon excitation from the ground state to a higher energy state [52]. Furthermore, we used 808 nm lasers at 1.5 to 2 W/cm^2^ for PDT/PTT, which is within the range of laser fluence rates used in previous studies for PDT in vitro and in vivo with an 808 nm laser [13,53,54]. 

### 3.4. Bio-Distribution and In Vivo Photothermal Effects

The ex vivo IVIS fluorescence imaging shows a strong fluorescence signal found exclusively in the brain tumor area of a tumor-bearing mouse immediately after CED of ILs (6 days post-implantation of U87MG cells), in contrast to PBS administration (Figure S5). Most importantly, ILs could be specifically retained in the brain tumor, without distribution to other major organs, 6 days after CED (12 days post-implantation of U87MG cells). The fluorescence intensity within the ROI of the explanted brain at the end of the observation period (day 12) was 72% of its initial value (day 6). Combining with the photostability of IR-780 in ILs in vitro, the long-term retention of ILs after CED to the brain endorses its use for consecutive laser irradiation. In comparison to IV delivery [55], CED could specifically deliver liposomal IR-780 to the brain for alleviating associated toxicity from delivered therapeutics, while simultaneously facilitating NIR imaging and improved PTT/PDT of glioma [56].

Considering the penetration of NIR into tissue, as the animals used in this study were nude mice with very thin skulls (~0.32 mm from transverse view of MRI), the light can penetrate the skull and reach the tumor from the same burr hole created during CED. This may be translated to clinical practice, as CED has been used in preclinical and clinical studies for treating glioblastoma [57]. Furthermore, using an internal laser source, by implanting a laser catheter into the tumor as in laser interstitial thermal therapy (LITT) for treating glioblastoma, may also be applicable after CED in the future clinically [58]. 

To confirm the in vivo photostability of ILs, the photothermal response of ILs in vivo was judged from real-time thermal images when mice receiving CED of PBS, free IR-780 or ILs were treated with NIR laser (up to 5 min) on day 7, 9 and 11 post-implantation of tumor cells (Figure S6). During the first laser treatment, mice in ILs and free IR-780 groups showed identical peak temperature profiles, with temperatures rising from 37 °C to 54 °C within 1 min, which stayed constant thereafter (Figure S6A). Administration of PBS led to negligible temperature changes after laser treatment, as expected. The mice in the IL group during second laser treatment showed a similar temperature profile as the first treatment. In contrast, mice in the free IR-780 group show reduced photothermal response with a drop of peak temperature from 54 °C to 44 °C (Figure S6B). During the third laser treatment, the IL group shows a slightly retarded rate of temperature rise but still manages to reach a peak temperature of 54 °C within 2 min. In contrast, the free IR-780 group showed drastically diminished photothermal effects, only to show a similar temperature profile as in the control PBS group (Figure S6C). Undoubtedly, such remarkable retention of in vivo photothermal responses of ILs over free IR-780 in vivo when exposed to successive laser treatment correlates well with the fast degradation and inferior photostability of IR-780 in vitro. Nonetheless, other than improved photostability, free drugs administered by CED showed early clearance from the central nerve system compared to drugs loaded within nanoparticles, which may also lead to rapid clearance of free IR-780 from the brain in contrast to long-term retention of ILs [59]. 

**Figure 8:**
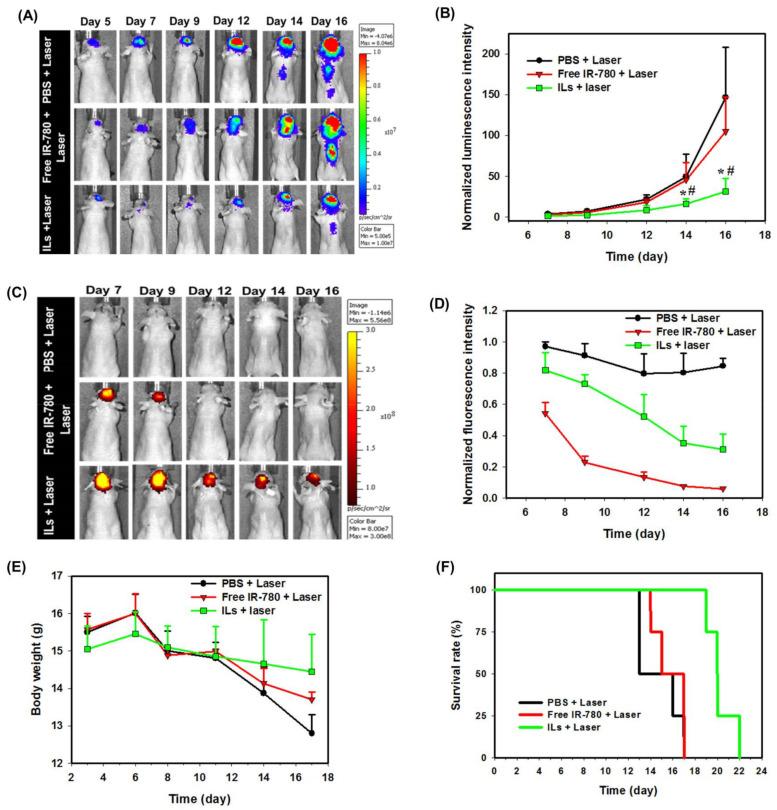
The representative images from bioluminescence imaging (BLI) (**A**) and the normalized bioluminescence intensity (normalized to day 5) (**B**) post-implantation of U87MG cells. The representative images from near infrared (NIR) fluorescence imaging (**C**) and the normalized fluorescence intensity (normalized to day 6) (**D**) post-implantation of U87MG cells. The change of body weight (**E**) and the animal survival rate (**F**) post-implantation of U87MG cells. * *p* < 0.05 compared with PBS + laser. ^#^ *p* < 0.05 compared with free IR-780 + laser.

The anti-tumor efficiency was followed from bioluminescence imaging (BLI) signal intensity with IVIS. The first BLI was carried out before CED on day 5 post-implantation of tumor cells, where similar intensity levels were noted in all three groups (Figure 8A). On day 7, after the first laser treatment, reduction of the BLI signal intensity was noted in ILs + laser and free IR780 + laser groups, due to the concurrent PTT/PDT of brain tumor xenografts. Increased BLI signal intensity was noticed in the PBS + laser group at the same time point. On day 9 after the second laser treatment, the BLI intensities increased in the free IR-780 + laser group and the PBS + laser group, but no change in BLI intensity was observed from the ILs + laser group. A similar trend was observed on day 12 after the third laser treatment on day 11, although no significant difference in BLI intensity among groups could be observed (Figure 8B). Nonetheless, significance was shown on day 14 and 16 with the ILs + laser group showing a significantly lower normalized luminescence intensity than other groups, suggesting only ILs can inhibit the growth of glioma cells under repeated laser treatment in vivo. To confirm this hypothesis, that anti-cancer efficacy is associated with the photostability of free IR-780 in vivo, NIR fluorescence imaging of treated mice was carried out, where drastic changes of signal intensity were noted only for free IR-780, especially after day 12 (Figure 8C). From quantitative comparison of NIR imaging results, the in vivo fluorescence signal intensity decreased immediately after the laser treatment for both free IR-780 and ILs on day 7, with normalized fluorescence intensity (normalized to day 6) being less than 1 (Figure 8D). This trend could be also observed after the second and third laser treatment on day 9 and 11. Nonetheless, it is clear that ILs could substantially improve the degradation of IR-780 due to NIR laser irradiation, showing 52% normalized fluorescence intensity on day 12, in comparison with 13% for free IR-780. Taken together, we successfully proved our hypothesis, that liposomal IR-780 (ILs) can enhance photostability of IR-780 in vivo, for improved PTT/PDT of brain tumor, as revealed from the significant reduction of intracranial tumor size from normalized BLI intensity (Figure 8B). 

From a safety perspective, the body weight of the mice was continuously monitored throughout the treatment period (Figure 8E). The body weight showed decreasing trends for all groups after day 6. Nonetheless, it is evident that mice in the ILs + laser group could better maintain their body weight than other groups to the end of the observation period, indicating fewer side effects from this treatment. To determine animal survival, we sacrificed the mouse when it lost more than 25% of the initial body weight or when it showed back hunk, hemiparesis or seizures. From the survival curve in Figure 8F, the median survival time of PBS + laser and free IR-780 + laser group was 16 days, while that for the ILs + laser group was 22 days. Furthermore, the survival time of the ILs + laser group (21.3 ± 1.1 days, mean ± SD) significantly increased over that of the PBS + laser group (15.8 ± 0.8 days) and the free IR-780 + laser groups (15.8 ± 0.3 days), with no significant difference found between the latter two groups. The significantly prolonged survival times as well as the reduced tumor sizes (Figure 8B) endorse a highly efficient treatment modality of brain tumors by concurrent PTT/PDT via CED of ILs.

### 3.5. MRI and PET/CT Studies 

**Figure 9:**
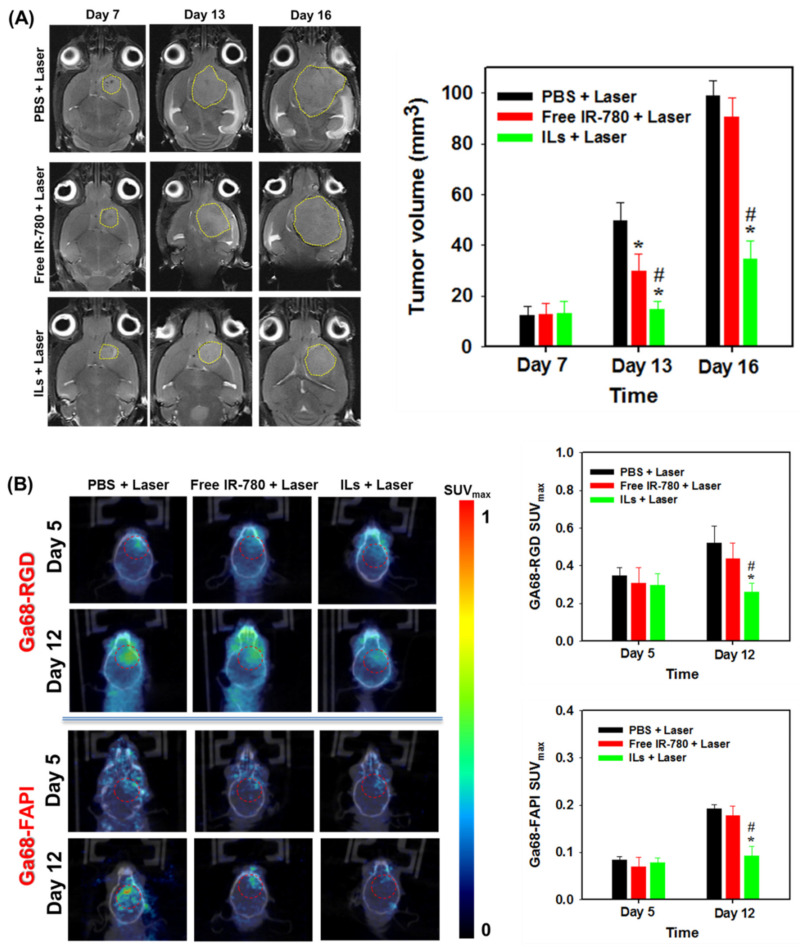
The representative coronal view images and the calculated tumor volumes from magnetic resonance imaging (MRI) (**A**), and Ga68-RGD and Ga68-FAPI positron emission tomography/computed tomography (PET/CT) (**B**) post-implantation of U87MG cells. * *p* < 0.05 compared with PBS + laser, ^#^ *p* < 0.05 compared with free IR-780 + laser.

As magnetic resonance (MR) imaging (MRI) is a paradigmatic method for diagnosis and identification of anatomical gliomas site [60], we used MRI at three different time points, i.e., on days 7, 13 and 16, for assessing the anti-tumor efficiency (Figure 9A). From ROI circled in yellow lines, no significant difference in tumor volume was found among all groups on day 7 (Figure 9B). However, differences in treatment efficacy were manifested on day 13 and 16 among all groups, with significantly reduced tumor volume shown from the ILs + laser group. The mean tumor volumes measured for the PBS + laser group were 49.5 mm^3^ and 99 mm^3^ on day 13 and 16, respectively. The free IR-780 + laser group showed some treatment benefits with 32.7 mm^3^ and 90.6 mm^3^ mean tumor volumes at the same time points. Most importantly, by retarding glioma growth to ~30% of the tumor size in the control, the ILs + laser treatment resulted in 14.8 mm^3^ (day 13) and 34.6 mm^3^ (day 16) mean tumor volume and provided the best anti-tumor outcomes, consistent with the prolonged survival time of glioma-bearing mice.

Angiogenesis is a naturally occurring physiological process that involves a complicated chemical process. This helps cancer cells for their growth and spread across various parts of the body or organ through chemical signals [61]. Angiogenesis is characterized by the expression of α_ν_β_3_ integrin in neo-vessel endothelial cells that binds specifically to Arg–Gly–Asp (RGD) peptide [62]. The expression of α_ν_β_3_ integrin is correlated with the malignancy of glioma, in conjunction with tumor-related angiogenic processes, to facilitate tumor progression [63]. Therefore, angiogenesis imaging with the help of radio-labelled RGD peptide may provide favorable results in locating tumors [64]. Among many radioisotopes, radionuclide 68Ga is a convenient option, as it is readily accessible and cost-effective. The 68Ga-based radiopharmaceuticals also display high spatial resolution compared to single-photon emission computed tomography, enabling more detailed quantification. In addition, with a 68 min half-life, 68Ga offers sufficient time for the preparation of small peptides or proteins [65]. The fibroblast activation protein (FAP) is overexpressed by many cancer-associated fibroblasts of several tumor entities, which are different from normal fibroblasts from the difference in their FAP specific expression. Therefore, targeting FAP with FAP-inhibitors (FAPI) is a new diagnostic approach allowing the visualization of tumor stroma, although they were first developed as anticancer drugs [66]. Radiolabeled FAPI (Ga68-FAPI) has been used for PET/CT molecular imaging and diagnosis, demonstrating a favorable biodistribution and high uptake in xenograft tumors as well as in patients with various malignancies with activated stromal fibroblasts [67]. Moreover, the first PET studies in human with Ga68-FAPI provided a high tumor-to-noise ratio imaging tool for identifying a broad range of tumors [68]. We therefore used PET/CT along with Ga68-RGD or Ga68-FAPI to study the treatment efficacy in the U87MG xenograft brain tumor model. As shown in Figure 9B, only the ILs + laser group showed a minimum change of signal intensity before treatment (day 5) and after treatment (day 12) from the coronal view PET/CT images. Most importantly, this group showed a significantly decreased maximum standardized uptake (SUV_max_) value obtained within the circled ROI for both Ga68-labeled RGD and FAPI, confirming ILs + laser treatment provides improved PTT/PDT to delay tumor growth, as shown from MRI. 

### 3.6. Histological Analysis 

**Figure 10:**
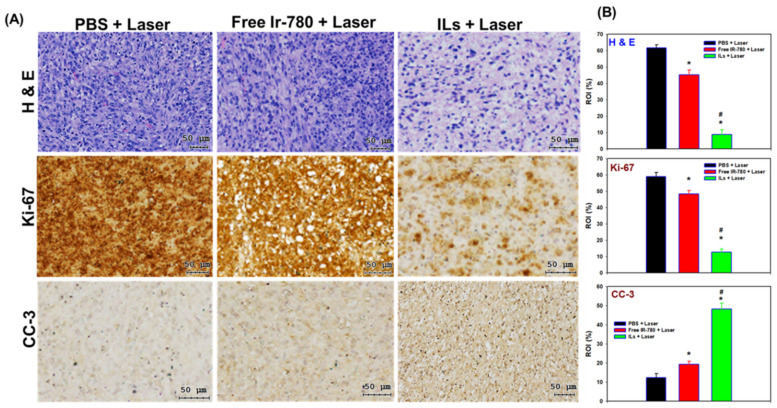
(**A**) The representative H&E staining, Ki-67 and cleaved caspase-3 (CC-3) immunohistochemical (IHC) staining images of paraffin-embedded tumor sections. (**B**) The corresponding positively stained area percentage within region of interest (ROI) (%) (n = 3). * *p* < 0.05 compared with PBS + laser. ^#^ *p* < 0.05 compared with free IR-780 + laser.

For histological analysis in Figure 10A, the brain tumor sections from H&E staining indicated that PBS + laser treatment does not develop detectable necrosis. Some necrosis areas were noted in the free IR-780 + laser group. In contrast, large areas of necrosis could be observed after ILs + laser treatment, as revealed from the excellent anti-tumor efficiency from BLI (Figure 8B). Follow-up image analysis using PAX-it software also showed significantly reduced cell nucleus area percentage within the ROI after ILs + laser treatment (Figure 10B). The IHC staining of cell proliferation marker (Ki-67) supports the H&E staining results. The area percentage of immunoreactive Ki-67 marker within ROI drastically reduced after ILs + laser treatment, to be ~26% (~22%) that of free IR-780 + laser (PBS + laser) treatment, indicating pronounced inhibition of growth of U87MG glioma cells (Figure 10B). Furthermore, a reverse trend of the immunoreactivity of the cell apoptotic marker, cleaved caspase-3 (CC-3), confirms that improved PTT/PDT could induce more cell apoptosis in the ILs + laser group. Indeed, the ILs + laser group showed 3.7-fold and 2.4-fold higher positively stained areas of CC3 compared to PBS + laser and free IR-780 + laser groups, due to the high cell apoptosis rate (Figure 10B).

To evaluate safety of the treatments, we conducted histology of major organs and blood analysis of sacrificed mice. We did not observe any apparent changes of the H&E staining results of major organs (Figure S7), revealing that IL delivery via CED as well as laser irradiation can avoid organ damage. From the results of hematological or biochemistry analysis of blood samples, we did not notice significant differences of all analyzed biochemical and hematological parameters in mice subject to ILs + laser treatment compared to PBS + laser as well as free IR-780 + laser groups, indicating no acute toxicity from the treatment (Table S1). 

## 4. Conclusions

By entrapping IR-780 within the lipid bilayer of cationic liposomes to maintain its photostability during successive NIR laser irradiation, we successfully prepared ILs in this study and demonstrated its improved PTT/PDT of intracranial glioma after CED. The application of ILs for PTT/PDT in vitro could be confirmed from real-time temperature change and ROS generation in vitro under NIR laser irradiation, which leads to cytotoxicity and apoptosis/necrosis of U87MG glioma cells. Compared to free IR-780, the intracellular uptake of ILs was substantially enhanced, and the IR-780 in ILs displayed enhanced photostability in vitro and in vivo after repeated laser irradiation for extending the PTT/PDT therapeutic efficacy. Using xenograft U87MG glioblastoma tumor models in mice brain, we demonstrate long-time retention of ILs in the brain tumor after CED, which facilitates PTT/PDT of intracranial glioma in vivo. Compared to free IR-780, ILs offers significant improvement of laser-assisted PTT/PDT in glioma treatment, judging from analysis with diagnostic imaging tools (BLI, MRI and PET/CT), as well as upregulation of apoptosis marker CC3 and downregulation of proliferation marker Ki-67 in tumor sections. Overall, the combination of CED of ILs with consecutive NIR laser treatment could provide an alternative approach for treating solid tumor in the brain.

## Data Availability

The data presented in this study are available on request from the corresponding author.

## Supplementary Materials

The following are available online at https://www.mdpi.com/article/10.3390/cancers13153690/s1, Figure S1: The timeline for establishing intracranial xenograft tumor model, convention-enhanced delivery (CED) and assessment of anti-tumor efficacy by bioluminescence imaging (BLI), magnetic resonance imaging (MRI), as well as positron emission tomography/computed tomography (PET/CT), Figure S2: The pictures showing the administration of samples into mouse brain via convention-enhanced delivery (CED), Figure S3: The ROS generation with DPBF probe due to NIR irradiation (1 W/cm^2^) of free IR-780 (A) and ILs (B) solution for 120 s. (C) The change of relative absorbance (A/A0) at 410 nm of free IR-780, ILs and PBS (control), Figure S4: The expression of heat shock protein 70 (HSP70) by U87MG cells from Western blot analysis by treating with PBS (control), IR-780 or ILs with or without NIR laser irradiation, Figure S5: The ex vivo NIR fluorescence imaging of explanted brain and major organs from tumor-bearing mice after CED of ILs or PBS (control) at different times post-implantation of U87MG cells. The fluorescence signal intensity within the ROI of the explanted brain is shown below the images, Figure S6: The representative real-time temperature distribution and the time-lapsed peak temperature profiles (mean ± SD, n = 4) of tumor-bearing nude mice. One, three and five days after convection-enhanced delivery (CED) of PBS, free IR-780 or ILs to tumor-bearing mice, the brain was irradiated with NIR laser (1 W/cm^2^) for 5 min, and thermal images were captured with an infrared camera during the 1st (A), 2nd (B) and 3rd (C) laser treatment, Figure S7: The H&E staining results of tissue sections from major organs of tumor-bearing mice after different treatments. Bar = 100 μm, Table S1: The hematological parameters and biochemistry analysis from treated mice.
