# Supplementary material for: Liposomal IR-780 as a Highly Stable Nanotheranostic Agent for Improved Photothermal/Photodynamic Therapy of Brain Tumors by Convection-Enhanced Delivery
Source: Cancers (Basel). 2021 Jul 22;13(15):3690. doi: 10.3390/cancers13153690 (PMC8345063; doi:10.3390/cancers13153690)
Supplement: Supplementary file 1 [file cancers-13-03690-s001.zip › cancers-1249585-supplementary.pdf]

Supplementary Material

Liposomal IR-780 as a Highly Stable Nanotheranostic Agent for Improved Photothermal/Photodynamic Therapy of Brain Tumors by Convection-Enhanced Delivery

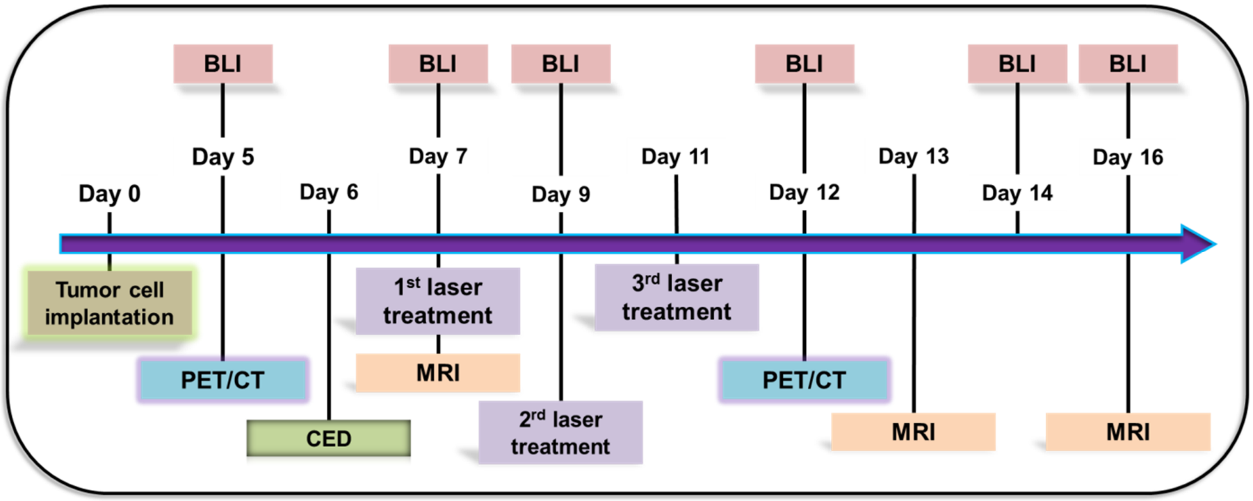

Figure S1. The timeline for establishing intracranial xenograft tumor model, convention-enhanced delivery (CED) and assessment of anti-tumor efficacy by bioluminescence imaging (BLI), magnetic resonance imaging (MRI), as well as positron emission tomography/computed tomography (PET/CT).

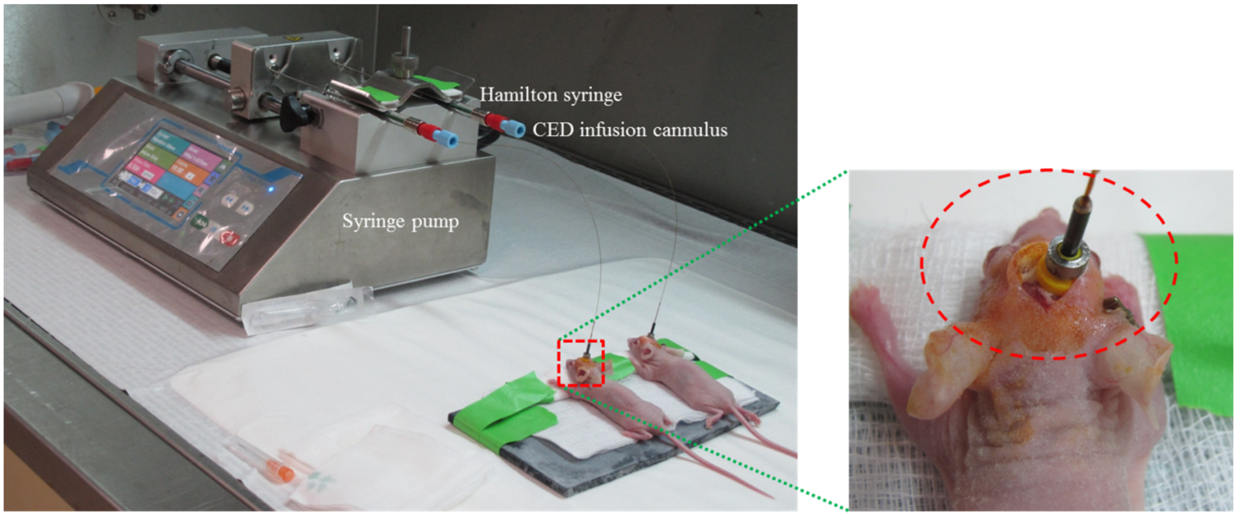

Figure S2. The pictures showing the administration of samples into mouse brain via convention-enhanced delivery (CED).

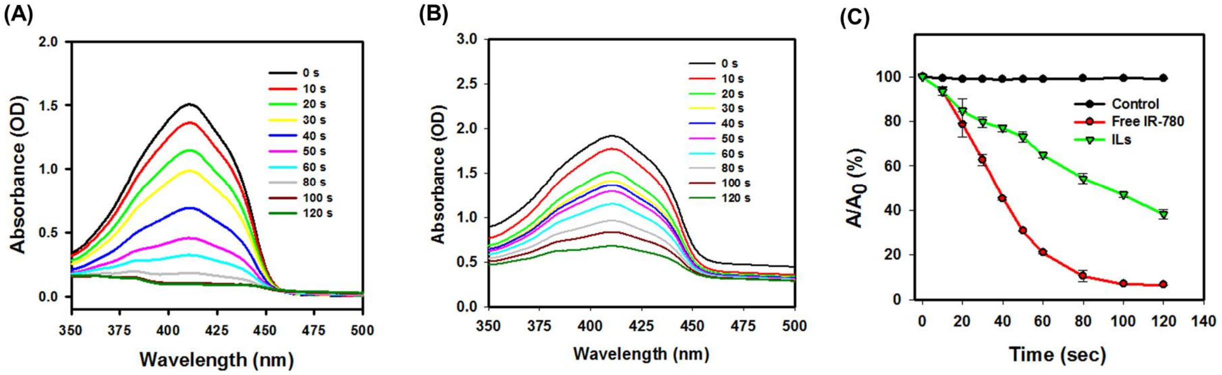

Figure S3. The ROS generation with DPBF probe due to NIR irradiation (1 W/cm^2) of free IR-780 (A) and ILs (B) solution for 120 s. (C) The change of relative absorbance (A/A_0) at 410 nm of free IR-780, ILs and PBS (control).

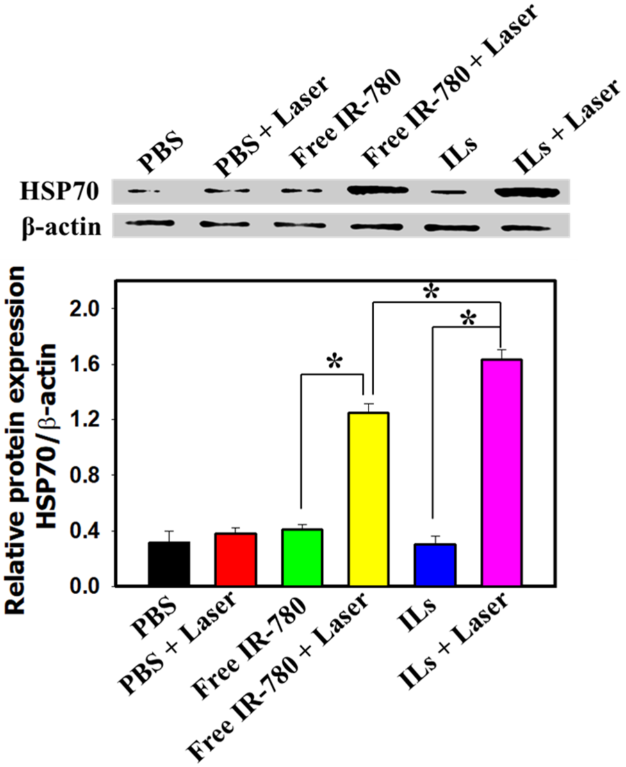

Figure S4. The expression of heat shock protein 70 (HSP70) by U87MG cells from Western blot analysis by treating with PBS (control), IR-780 or ILs with or without NIR laser irradiation.

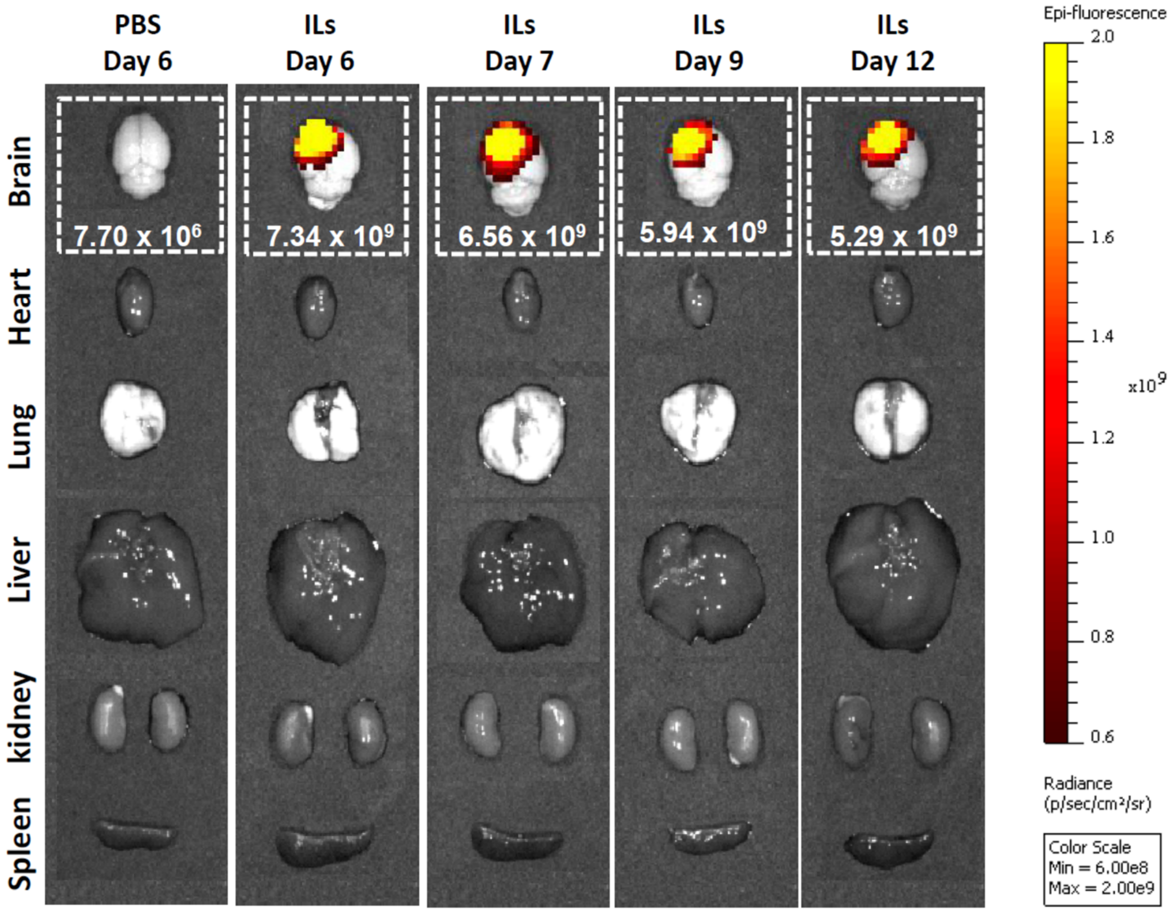

Figure S5. The ex vivo NIR fluorescence imaging of explanted brain and major organs from tumor-bearing mice after CED of ILs or PBS (control) at different times post-implantation of U87MG cells. The fluorescence signal intensity within the ROI of the explanted brain is shown below the images.

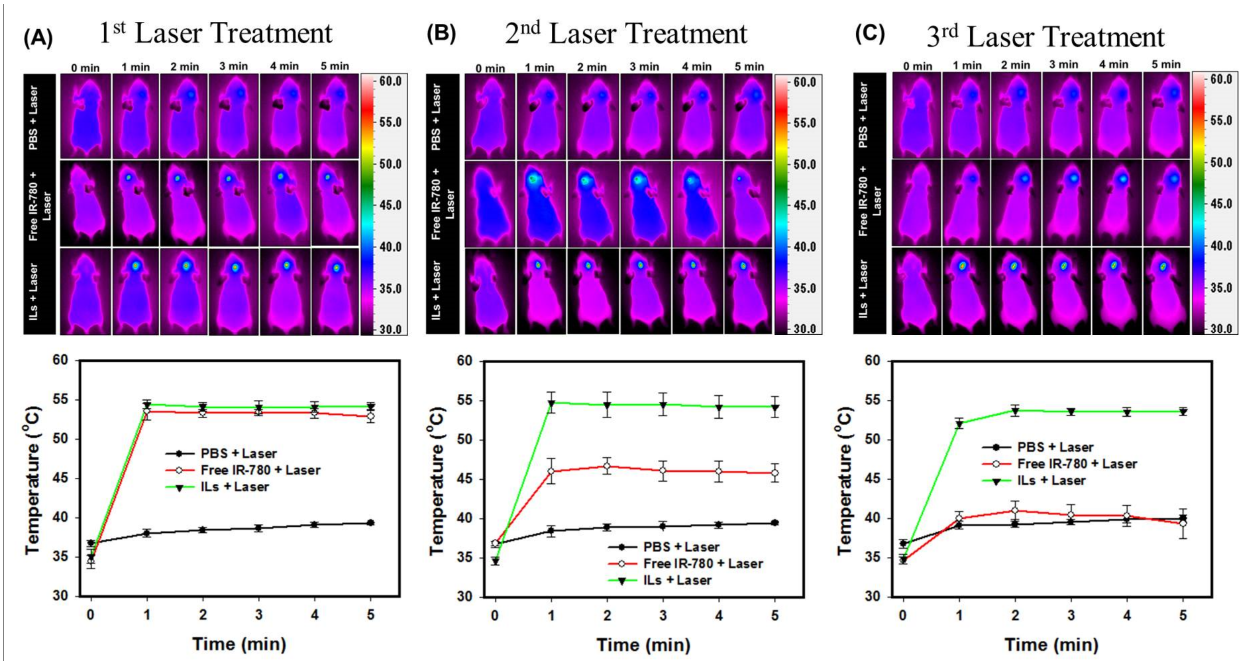

Figure S6. The representative real-time temperature distribution and the time-lapsed peak temperature profiles (mean ± SD, n = 4) of tumor-bearing nude mice. One, three and five days after convection-enhanced delivery (CED) of PBS, free IR-780 or ILs to tumor-bearing mice, the brain was irradiated with NIR laser (1 W/cm^2) for 5 min and thermal images were captured with an infrared camera during the 1st (A), 2nd (B) and 3rd (C) laser treatment.

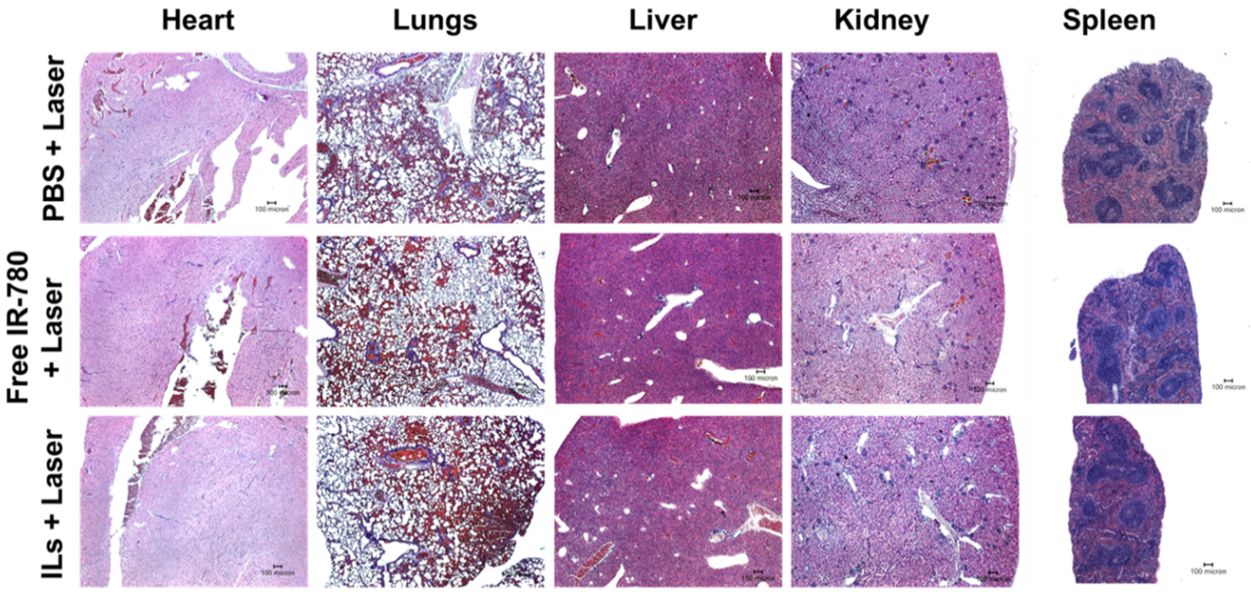

Figure S7. The H&E staining results of tissue sections from major organs of tumor-bearing mice after different treatments. Bar = 100 μm.

Table S1. The hematological parameters and biochemistry analysis from treated mice.

Items	**PBS + Laser**	**Free IR-780 + Laser**	**ILs + Laser**
WBC (10^9/L)	5.76 ± 2.66	8.86 ± 3.98*	4.63 ± 2.77$^{*,\#}$
RBC (10^{12}/L)	8.18 ± 0.05	8.45 ± 0.41*	8.82 ± 0.66$^{*,\#}$
Platelet (10^9/L)	133.13 ± 12.54	141.33 ± 11.01*	136.13 ± 9.16$^{*,\#}$
Hemoglobin (g/L)	972 ± 86.6	860 ± 112.2*	1024 ± 156.5$^{*,\#}$
AST (IU/L)	86 ± 8.9	80 ± 11.2*	93.7 ± 16.5$^{*,\#}$
ALT (IU/L)	129 ± 10.67	122 ± 18.1*	137 ± 23.73$^{*,\#}$
BUN(mmol/L)	43.5 ± 4.5	40 ± 2.44*	40.33 ± 5.4$^{*,\#}$

WBC, white blood cells; RBC, red blood cells, AST, aspartate aminotransferase; ALT, alanine aminotransferase, BUN, blood urea nitrogen. * $p > 0.05$ compared with PBS + Laser, $^{\#}$ $p > 0.05$ compared with free IR-780 + Laser.
